# Genome-wide exploration of biosynthetic gene clusters and their association with virulence in the entomopathogenic fungus *Beauveria*

**DOI:** 10.1007/s10142-026-01958-1

**Published:** 2026-06-30

**Authors:** Robson dos Santos Soares, Alexandra de Azevedo da Rocha, Matheus Camargo, Maria Eduarda Deluca João, Augusto Schrank, Henrique R.M. Antoniolli, Charley Christian Staats

**Affiliations:** 1 Programa de Pós-Graduação em Biologia Celular e Molecular, Centro de Biotecnologia, Universidade Federal do Rio Grande do Sul, Av. Bento Gonçalves 9500, Porto Alegre, 91501-970 Agronomia, RS Brasil; 2 National Institute of Science and Technology in Human Pathogenic Fungi, São Paulo, Brazil

**Keywords:** *Beauveria*, Phylogenomics, Biosynthetic gene clusters (BGCs), Virulence factors, Secondary metabolites, Comparative genomics.

## Abstract

Species of the genus *Beauveria* are widely used as biological control agents due to their ability to infect and kill a broad range of arthropod pests. Despite their agricultural importance, the genomic diversity underlying virulence and secondary metabolism across the genus remains uncharacterized. In this study, we performed a comprehensive phylogenomic and biosynthetic gene cluster (BGC) analysis of 322 high-quality *Beauveria* genomes, including one newly sequenced *Beauveria bassiana* isolate (AS272) obtained from soil in southern Brazil. The genome of AS272 comprises 32.9 Mb with 9,778 predicted genes and high completeness (96.7% BUSCO). Comparative analysis revealed extensive conservation of orthogroups but indicated that the increased gene repertoire of AS272 is primarily associated with the expansion of existing gene families, particularly transporters belonging to the major facilitator superfamily (MFS) and ATP-binding cassette (ABC) families. Phylogenomic reconstruction based on single-copy orthologs resolved major species clades within the genus and identified potential misidentified genomes in public databases. Genome mining using antiSMASH identified 14,160 BGCs across the dataset, with NRPS and PKS clusters dominating the biosynthetic landscape. Clustering with BiG-SCAPE revealed a mixture of highly conserved gene cluster families (GCFs) and numerous lineage-specific clusters, highlighting both evolutionary stability and diversification of secondary metabolism within *Beauveria*. Notably, *B. bassiana* lacked strictly conserved core GCFs across all isolates, instead exhibiting a heavy-tailed distribution of cluster frequencies consistent with substantial intraspecific genomic plasticity. Protein–protein interaction network analysis further indicated that several conserved BGCs are embedded within networks enriched for proteins associated with host–pathogen interactions. Together, these findings reveal a dynamic genomic architecture underlying secondary metabolism and pathogenic potential in *Beauveria*, providing a comparative framework for understanding virulence evolution and identifying candidate pathways for future functional and biotechnological studies.

**Supplementary Information:**

The online version contains supplementary material available at 10.1007/s10142-026-01958-1.

## Introduction

*Beauveria* is a genus of entomopathogenic fungi widely recognized for its use in the biological control of arthropod pests (de Sousa et al. 2025). Members of this genus infect their hosts using a common mechanism involving adhesion and germination of conidia on the insect cuticle, followed by penetration, internal colonization, and host death (Hong et al. 2024). Compared to conventional chemical insecticides, *Beauveria* species exhibit a narrower host range, lower environmental impact, and are considered safe for non-target organisms, including humans, animals, and plants (Mascarin and Jaronski 2016). These features support the development and commercialization of various *Beauveria*-based biopesticide formulations, which are applied in integrated pest management (IPM) strategies across different agricultural systems (Andreata et al. 2025). In Brazil, by the beginning of 2026, a total of 160 biocontrol products containing *Beauveria* sp. had been registered, highlighting its effectiveness in controlling insect pests (Ministério da Agricultura e Pecuária 2026).

The commercial importance of the genus *Beauveria* reflects its high pathogenic potential, which is supported by the diversity of molecules produced during the infection cycle, such as insecticidal toxins, immunosuppressive compounds, and antimicrobial metabolites (Oberti et al. 2025; Farag et al. 2025; Aly et al. 2025). Secondary metabolites are also produced during host infection (Chaithra et al. 2024), including oosporein, bassianolide, and beauvericin, which play pivotal roles in virulence by promoting host immune suppression, tissue colonization, and insect mortality (Wang et al. 2021). These compounds belong to distinct classes of secondary metabolites, including polyketides and nonribosomal peptides or depsipeptides, which are encoded by well-defined biosynthetic gene clusters (BGCs) comprising core synthase genes, such as polyketide synthases (PKSs) and nonribosomal peptide synthetases (NRPSs), along with tailoring enzymes, transporters, and regulatory elements (Keller 2019). The profile and production of these compounds vary among species and strains, highlighting the genomic plasticity of the genus and its ability to adapt to different hosts and environments (Solano-González et al. 2023). In other fungal genera, comparative genomic analyses have shown that gene annotation and prediction of biosynthetic gene clusters (BGCs) associated with secondary metabolites are broadly distributed among species, representing a promising approach for investigating molecular mechanisms of virulence based on genomic data (Valero-Jiménez et al. 2016). Genomic plasticity contributes to diversification across species, yet core genes associated with pathogenicity, such as those involved in host adhesion, cuticle degradation, and stress tolerance, are commonly conserved among phylogenetically related species, highlighting their essential role in infective capacity and survival (Wang and Wang 2017).

Despite advances in genome sequencing technologies and data availability, the lack of standardized curation and robust functional annotation in public databases continues to limit the effective use of genomic information in comparative studies. Rigorous curation of genomic datasets, including reannotation and functional validation of genes, is therefore essential to ensure the reliability of analyses aimed at elucidating metabolic diversity, virulence determinants, and evolutionary processes, particularly in entomopathogenic fungi (Wang and Wang 2017). Here, we conducted a comprehensive phylogenomic and biosynthetic gene cluster (BGC) analysis of more than 300 high-quality *Beauveria* genomes, including one newly sequenced by our group. Our approach integrates taxonomic profiling, BGC classification, and the functional association of secondary metabolite biosynthesis with virulence-related features in the newly sequenced strain. Together, our findings provide insights into the evolutionary conservation, diversification, and pathogenic potential encoded within the genomes of this agriculturally important fungal genus.

## Materials and methods

### Genome sequencing, assembly, and annotation

The *B. bassiana* AS272 strain was obtained from soil in the state of Rio Grande do Sul, Brazil. The isolation process used suspension of soil samples in sterile water, followed by plating on Oatmeal Agar-CTAB medium supplemented with 50 µg/mL chloramphenicol. The pathogenicity of the isolates was confirmed by infection assays using the model arthropod *Tenebrio molitor*. The AS272 strain was maintained at 28 °C on PDA medium, and identification was performed by sequencing the B locus intergenic region (Bloc) (accession number: PQ220121.1), primers – B51-F 5′-CGACCCGCCAACTACTTTGA-3′ and B31-R 5′-GTCTTCCAGTACCACTACGCC-3′. (Camargo, unpublished data). For DNA isolation, 3-day-old mycelia were used, following the CTAB method (Dar et al. 2025) with a bead beater (FastPrep^®^−24 5G - MP Biomedicals). Sequencing was performed in a paired-end mode (2 × 150 bp) using an Illumina NextSeq 2000 after library preparation by LacTAD (https://www.lactad.unicamp.br/).

Prior to assembly, raw sequencing reads were subjected to quality filtering using fastp v 0.23.4 (Chen et al. 2018) under default parameters. *De novo* genome assembly was subsequently performed using SPAdes v 4.2.0 (Bankevich et al. 2012) under default parameters. To enhance assembly contiguity, the resulting contigs were scaffolded with RagTag v 2.1.0 (Alonge et al. 2022), utilizing the *Beauveria bassiana* ARSEF 2860 reference genome (NCBI RefSeq: GCF_000280675.1) as a guide. The final genome sequence has been deposited in the NCBI database under the accession number GCA_052426205.1. The assembled and scaffolded genome was processed using Funannotate v 1.8.1 (Palmer and Stajich 2020) to perform FASTA header standardization, sequence sorting, and repeat masking. Gene prediction was subsequently conducted utilizing BRAKER3 v 3.0.6 (Gabriel et al. 2024), with the *B. bassiana* ARSEF 2860 reference proteome serving as the training dataset. Finally, the resulting GFF3 file was integrated back into the Funannotate pipeline to finalize the functional annotation. Secondary metabolite biosynthetic gene clusters (BGCs) were subsequently predicted from the BRAKER3 outputs (FASTA and GFF3 formats) utilizing antiSMASH v8.0.4 (Blin et al. 2025) configured in the fungal mode. Gene family expansion was determined using CAFE 5 (v 1.1) (Mendes et al. 2021).

### ***Beauveria*** genome sequences

A total of 337 assembled genomes, deposited as belonging to the genus *Beauveria* were retrieved from the NCBI database using NCBI datasets v 18.6.0 (O’Leary et al. 2024) and assessed for completeness using the BUSCO 5.8.2 tool (Benchmarking Universal Single-Copy Orthologs) (Manni et al. 2021) with the orthologous genes dataset of Hypocreales (version 12). For phylogenomic analysis, all genome sequences were used, while for functional prediction, only those with BUSCO completeness scores above 90% were subsequently processed for gene prediction and functional annotation as described above using the Funannotate 1.8.1 (Palmer and Stajich 2020), as previously used for *Fusarium* studies (Gomez-Chavarria et al. 2024). The full list of accession codes, genome assembly statistics, and respective BUSCO completeness results are provided in Table S1.

## Phylogenomic analyses

Phylogenomic analyses were performed using the BUSCO_phylogenomics pipeline (Waterhouse et al. 2018) (https://github.com/jamiemcg/BUSCO_phylogenomics), which generates phylogenomic matrices based on single-copy orthologous genes. The pipeline uses output files from BUSCO (Manni et al. 2021) to identify conserved single-copy genes, which are aligned with MAFFT (Katoh and Standley 2013). Nucleotide sequences of each orthologous group were aligned and concatenated into a supermatrix, with one partition considered per gene. Phylogenetic inference was conducted using IQ-TREE 2.1.4-beta (Minh et al. 2020), applying the TESTMERGE model to automatically merge partitions with compatible substitution models. Model selection was performed automatically by ModelFinder based on the Bayesian Information Criterion (BIC). Phylogenetic support was estimated using ultrafast bootstrap approximation (UFBoot) with 1,000 replicates. The final phylogenetic tree was constructed exclusively with nucleotide data and included all 339 genomes, with two *Metarhizium* species used as the outgroup.

## Secondary metabolite gene cluster analysis

Gene cluster analysis was conducted using the local version of antiSMASH 8.0.4 (Blin et al. 2025). The classification of biosynthetic gene clusters (BGCs) into gene cluster families was carried out using BiG-SCAPE 2 (Biosynthetic Gene Similarity Clustering and Prospecting Engine) (Navarro-Muñoz et al. 2020). BGCs were grouped based on sequence similarity across a range of cutoff thresholds from 0.1 to 0.9. Clustering at a cutoff value of 0.6 provided an appropriate resolution for distinguishing BGC families. BGCs could be matched to previously characterized clusters by integrating the BiG-SCAPE algorithm with the Minimum Information about a Biosynthetic Gene cluster (MIBiG) database (Zdouc et al. 2025).

## Virulence association prediction

To investigate the potential association between BGCs and virulence, the predicted protein-coding genes within selected BGCs were queried against the PHI-base 4.18 (Urban et al. 2025) database using a local BLASTP 2.12.0 (Camacho et al. 2009). BLASTP hits derived from fungal species and exhibiting ≥ 50% amino acid sequence identity over ≥ 50% query coverage were considered putative virulence-associated homologs. This threshold was selected as a conservative criterion to balance sensitivity and specificity in homology-based virulence prediction and is consistent with previous fungal comparative genomics studies (Kuan et al. 2016; Looi et al. 2017; Marcet-Houben et al. 2021). Protein–protein interaction (PPI) analysis was performed using the STRING v12.0 database (Szklarczyk et al. 2023). Putative virulence determinants were queried against a custom STRING database generated by the “Annotate your proteome” workflow, in which the entire predicted proteome was automatically mapped to STRING orthologs using the platform’s internal sequence similarity and orthology assignment procedures under default settings. (available at https://version-12-0.string-db.org/organism/STRG0A07FVB).

## Material availability

The codes used for shell processing, as well as for R analysis and figures plotting, are available as Supplementary Files 1 and 2, respectively. Processing files are available at Zenodo (10.5281/zenodo.20672312).

## Results

### Characterization of ***B. bassiana*** AS272 genome sequence

**Fig. 1:**
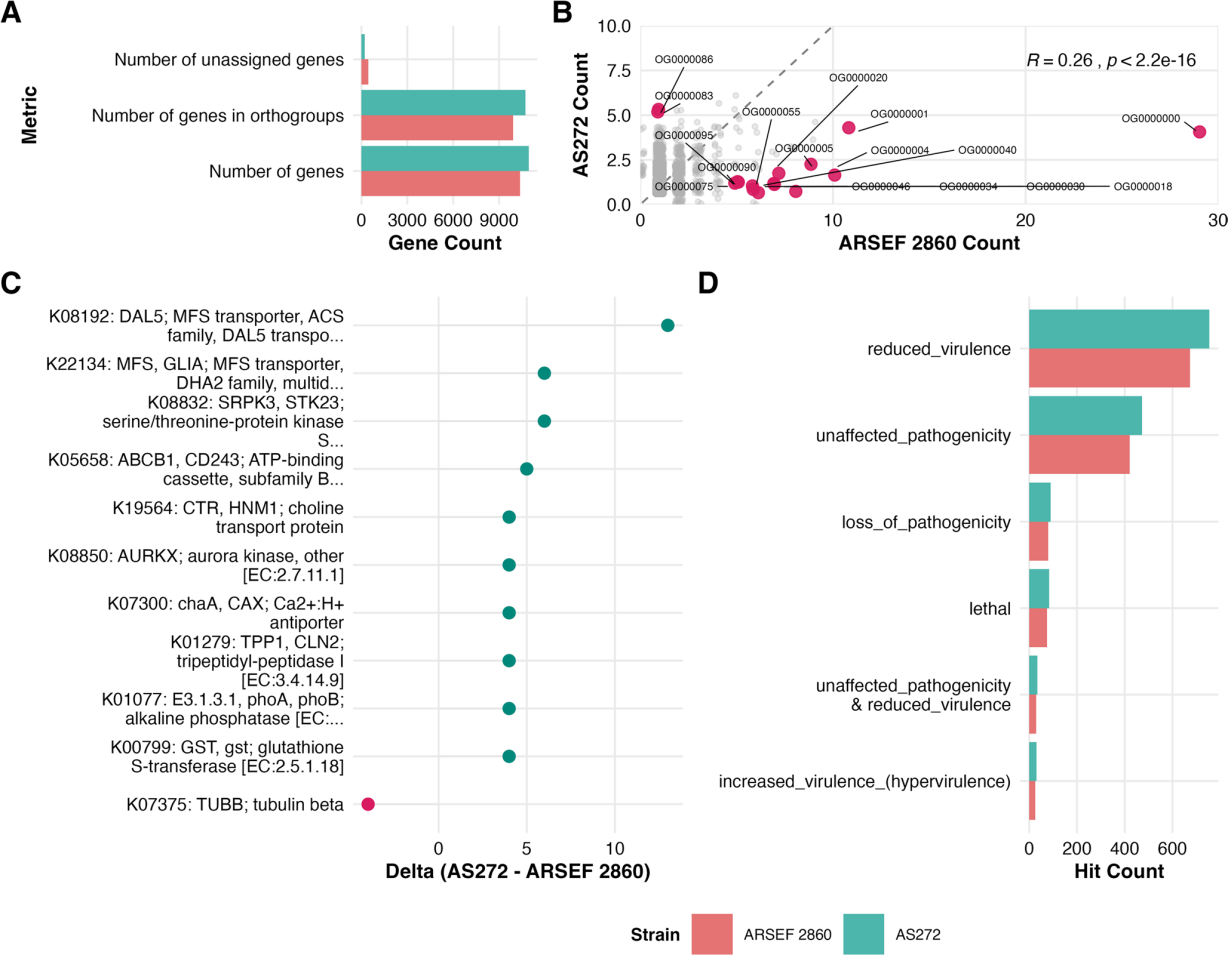
**Comparative genomic analysis of** ***B. bassiana*** **AS272 with the reference strain ARSEF 2860.** (**A**) Orthology and Proteome Metrics. Bar chart comparing the total predicted genes, genes assigned to orthogroups, and unassigned genes for AS272 and ARSEF 2860. (**B**) Orthogroup Gene Family Expansion. Scatterplot comparing gene counts per orthogroup between the two strains. (**C**) Functional Divergence of KEGG Orthologs (KO). Distribution of the most expanded and reduced functional categories in AS272. The horizontal axis represents the copy number delta (AS272 - ARSEF 2860). (**D**) Pathogenicity-Related Factors (PHI-base). Distribution of genes mapped to the Pathogen-Host Interactions database

To characterize the genomic characteristics of the high-virulence *B. bassiana* isolate AS272, a comparative genomics approach was employed, using the NCBI reference strain *B. bassiana* ARSEF 2860 as a control. The assembly of isolate AS272 comprises 32,999,432 bp with a high level of completeness (96.7% complete BUSCOs). BRAKER3 prediction identified 9,778 protein-coding genes, leading to 10,937 predicted proteoforms, which is slightly higher compared to reference (10,364 predicted proteoforms). OrthoFinder analysis revealed a high degree of conservation, with 8,984 orthogroups shared between the two isolates, representing 99.5% of the total orthogroup repertoire. Notably, isolate AS272 exhibited a significantly higher percentage of genes assigned to orthogroups (98.1%; 10,730 genes) compared to the reference strain (95.7%; 9,921 genes). Furthermore, AS272 showed a marked reduction in unassigned “singleton” genes (207; 1.9%) relative to ARSEF 2860 (443; 4.3%), suggesting that the increased gene count of AS272 is driven by the expansion of existing gene families (Fig. 1A).

A comparative analysis of gene family evolution identified 16 orthogroups (Table S2) with statistically significant quantitative differences (FDR-corrected p-value < 0.05) between the two isolates, as identified by CAFE 5. While the global orthogroup distribution remains broadly correlated (*R* = 0.26, *p* < 2.2e–16), pronounced lineage-specific expansions were observed in both genomes (Fig. 1B). Notably, families OG0000000 and OG0000001 showed the most substantial expansion in the reference strain ARSEF 2860 compared to AS272. Functional annotation via CDD search revealed that OG0000000 is predominantly composed of proteins containing the hAT family transposase domain (Dimer_Tnp_hAT, pfam05699), which are generally associated with mobile genetic elements and genomic plasticity. Furthermore, OG0000001 is enriched with members of the MRP_assoc_pro superfamily (cl33195) and ABC transporter domains (cl38913), which are essential for the regulated efflux of metabolites and xenobiotics. Conversely, the AS272 strain exhibited its own distinct, significant expansions, most notably in orthogroups OG0000083 and OG0000086. Annotation of OG0000086 revealed the presence of the RICTOR_N domain (pfam14664), a conserved structural component associated with the Target of Rapamycin (TOR) signaling complex, suggesting potential strain-specific adaptations in cellular growth regulation or environmental stress response in AS272.

Functional mapping via KEGG identified specific KEGG Orthologs (KO) with marked copy-number variation. The most significant expansion was observed in K08192 (an MFS transporter), which displayed a delta of 13 copies compared to the reference. This was followed by K22134 (MFS transporter), K08832 (serine-threonine kinase), and K05658 (ATP-Binding cassette). Conversely, a reduction was noted in K07375 (Tubulin beta), suggesting subtle structural or regulatory shifts relative to the reference strain (Fig. 1C). Finally, isolate AS272 displays a robust increase of pathogenicity-related factors mapped against the PHI-base database using stringent criteria (minimum of 50% identity over at least 50% of the length of the subject). Compared to ARSEF 2860, the strain AS272 exhibits a higher number of homologs linked to the “reduced virulence” and “loss of pathogenicity” categories, which points toward a potentially redundant set of virulence proteins (Fig. 1D). These functional enrichments point toward specialized adaptations in nutrient transport and environmental sensing that likely support the infection phenotype of this soil-derived isolate.

## Phylogenomic analyses

To establish the precise taxonomic identity and evolutionary context of the high-virulence isolate *B. bassiana* AS272, we performed a phylogenomic analysis using a maximum-likelihood approach. The phylogeny was reconstructed from a supermatrix of 247,446 bp derived from 204 genomic partitions, which represent 4,333 single-copy orthologs identified via BUSCO. The dataset included 339 genome sequences, comprising 337 *Beauveria* genomes (queried from NCBI as of March 2023) and two *Metarhizium* strains utilized as outgroups.

**Fig. 2:**
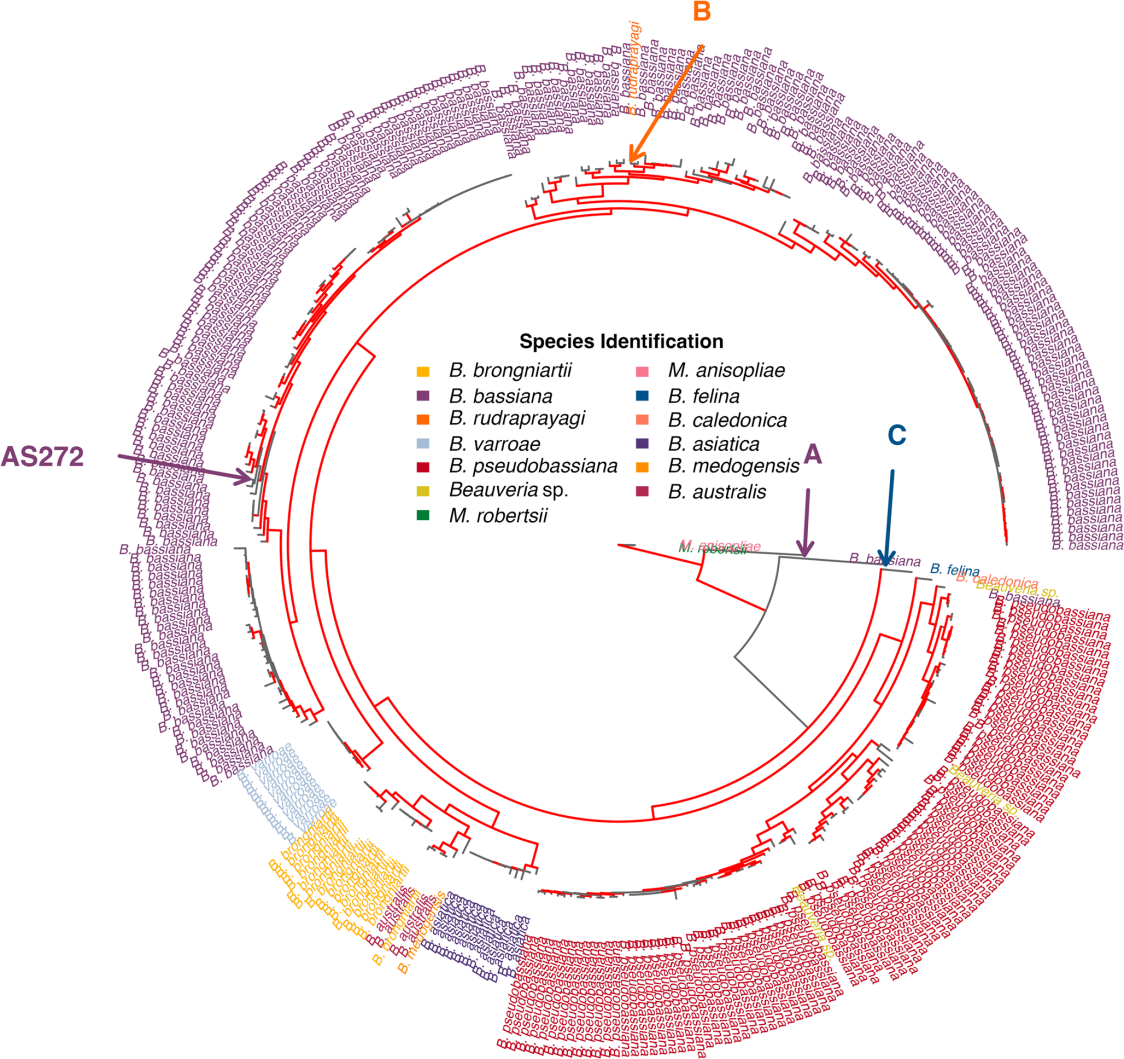
**Maximum Likelihood radial phylogeny of the genus** ***Beauveria***. Maximum-likelihood phylogeny inferred from a concatenated supermatrix of 247,446 bp across 204 genomic partitions, representing 339 isolates (337 *Beauveria* isolates and two *Metarhizium* outgroups). The tree was rooted using *M. robertsii* ARSEF 23 and *M. anisopliae* JEF-290. Branch colours indicate nodal support: internal branches subtending clades with ultrafast bootstrap (UFBoot) support ≥ 80% are shown in red, while weakly supported internal branches (UFBoot < 80%) and all terminal branches are shown in grey; terminal branches are uncoloured because bootstrap support is defined for internal nodes (clades) and not for individual tips. Tip labels are coloured by species identification as shown in the legend. For visualization only, branch lengths b were rescaled as log₁₀(√b + 1) to compress long branches and improve the legibility of densely clustered clades in the radial layout; this rescaling is strictly monotonic and therefore does not alter the topology or support values. Branch lengths shown are consequently not proportional to evolutionary distance and should not be interpreted quantitatively; the tree with untransformed maximum-likelihood branch lengths is provided as Figure S1. The purple arrow marks the position of *B. bassiana* isolate AS272; arrows labelled A, B, and C indicate phylogenetic incongruences discussed in the text

The resulting fan phylogeny resolved the genus into several major clades (Fig. 2). To clarify evolutionary distances within dense terminal radiations, branch lengths were visualized using a logarithmic square-root transformation. The most expansive lineage is the *B. bassiana* clade, which contains the majority of investigated isolates. Highly supported sister clades, which are indicated by red internal branches (≥ 80% UFBoot), were clearly delineated for *B. pseudobassiana*, *B. asiatica*, *B. varroae*, and *B. brongniartii*. Additionally, a distinct clade was recovered pairing *B. australis* with *B. medogensis*. The tree remains rooted by a robust outgroup containing *M. robertsii* and *M. anisopliae*. Notably, *B. bassiana* AS272 (GCA_052426205.1) was positioned within a well-supported subclade composed exclusively of *B. bassiana* strains (Fig. 2 - arrow AS272). This placement confirms its taxonomic identity and definitively separates it from sister species within the complex, such as *B. pseudobassiana* and *B. brongniartii*.

Despite the overall robustness of the topology, several phylogenetic discrepancies were identified. *B. bassiana* MBC618 branched entirely outside the *Beauveria* genus, clustering with the *Metarhizium* outgroup with absolute support (100% UFBoot/100% SH-aLRT; Fig. 2 Arrow A). This significant incongruence suggests a potential misidentification or genomic sequence contamination for this specific isolate. Furthermore, *B. rudraprayagi* MTCC8017 was nested deeply within the *B. bassiana* clade, grouping directly with *B. bassiana* ARSEF 2597 (Fig. 2 - Arrow B). This finding supports the hypothesis that *B. rudraprayagi* may represent a variant of *B. bassiana* rather than a distinct phylogenetic species. Similarly, *B. felina* SYSU-MS7908 appeared phylogenetically distant from all other represented taxa, occupying an isolated basal position (Fig. 2 - Arrow C). Collectively, these discrepancies highlight the critical need for rigorous phylogenomic validation to address the incidence of misidentified genomes and cryptic diversity currently present in public repositories.

### Conservation of BGCs among ***Beauveria*** genus analyses

**Fig. 3:**
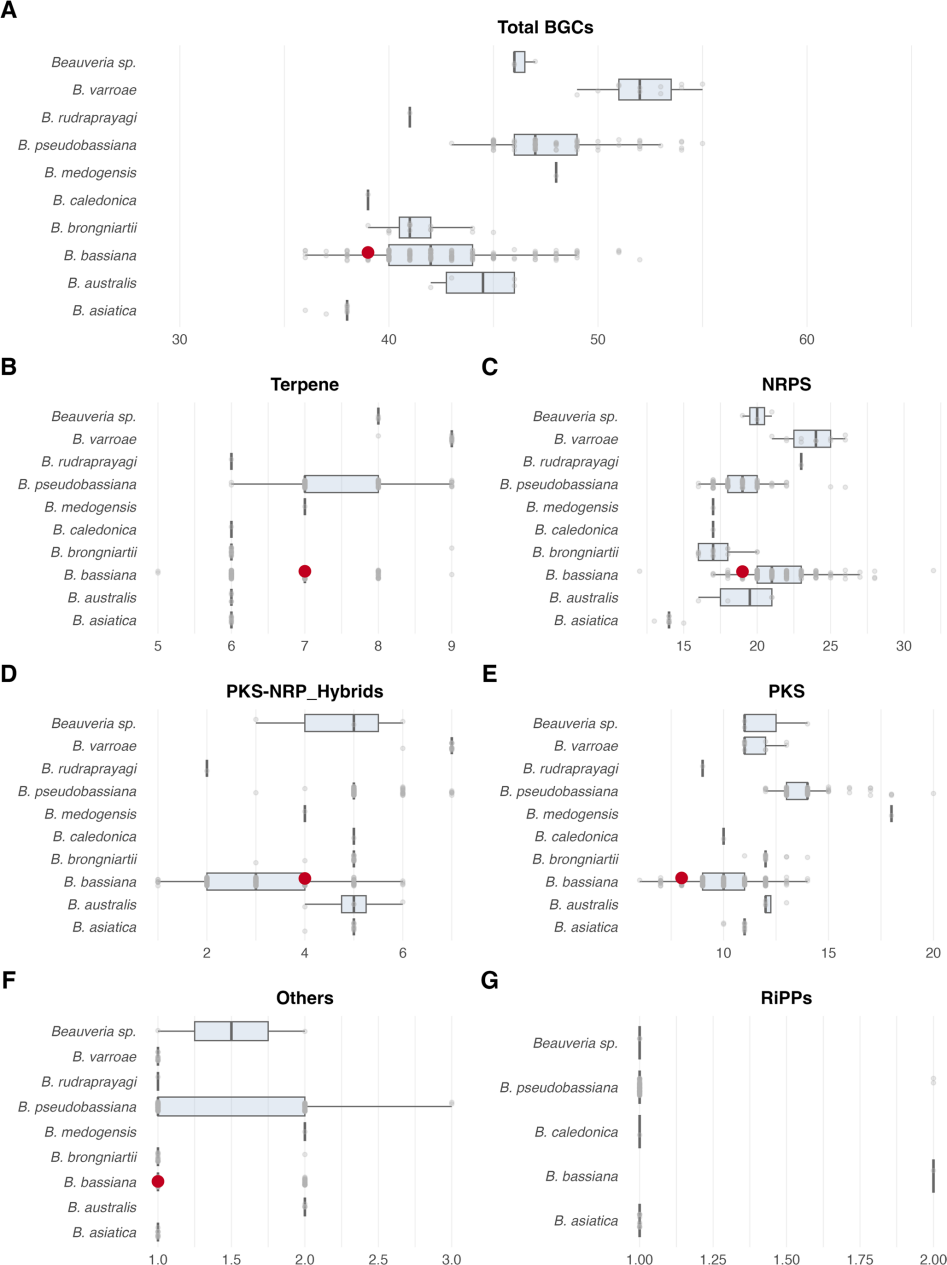
**Distribution and diversity of Biosynthetic Gene Clusters (BGCs) across the genus** ***Beauveria***. Comparative analysis of BGC abundance across 322 genomes representing 10 taxonomic groups. The top panel illustrates the total BGC count per genome (**A**), followed by stratification by chemical class: Terpene (**B**), Non-ribosomal Peptide Synthetase (NRPS - **C**), Polyketide Synthase-Non-ribosomal Peptide Hybrids (PKS-NRP Hybrids - **D**), Polyketide Synthase (PKS - **E**), Others (unclassified clusters - **F**), and Ribosomally synthesized and Post-translationally modified Peptides (RiPPs - **G**). Box plots represent the interquartile range (IQR), with the central line indicating the median and whiskers extending to 1.5 × IQR. Individual genomic data points are overlaid as semi-transparent blue circles to illustrate intra-species variation. The red circle in each panel specifically highlights the BGC counts for the high-virulence isolate *B. bassiana* AS272

With the taxonomic identity and evolutionary relationships of *B. bassiana* AS272 established, our focus shifted toward the functional genomic elements potentially associated with its virulence phenotype. Entomopathogenic fungi rely on a highly specialized set of secondary metabolites to facilitate host infection, evade insect immune responses, and compete in complex ecological niches. Therefore, the specialized biosynthetic capacity of AS272 was inferred and compared to those observed in the broader evolutionary history of the genus. To accurately reflect the current state of public databases, all genomes displaying a BUSCO completeness score above 90% were annotated using fungiSMASH, including the above phylogenetically incongruent isolates. Across the 322 genomes that passed this objective filter, a total of 14,160 BGCs were predicted, revealing a high degree of biosynthetic diversity and inter-specific variation within the genus (Fig. 3). The total number of BGCs per genome ranged from approximately 35 to 55, with *B. varroae* and *B. pseudobassiana* exhibiting the most expansive biosynthetic repertoires, maintaining medians above 45 clusters per genome. Conversely, considering the content of BGCs, the genomes of *B. asiatica*,* B. caledonica* and *B. rudraprayagi* appeared more compact (Fig. 3A). Even genomes with high fragmentation, as assessed by their N50 values (Table S1), displayed a number of BGCs that fall within the interquartile range of the genus. Therefore, the observed variation represents a bona fide unequal distribution of BGCs across *Beauveria* genomes, rather than an artifact of genome quality.

The distribution of Biosynthetic Gene Clusters (BGCs) by chemical class highlights a clear dominance of Non-Ribosomal Peptide Synthetases (NRPS) and Polyketide Synthases (PKS) across all species (Fig. 3B and G). NRPS BGCs represent the most abundant category identified (Fig. 3C). Notably, *B. varroae* exhibited a significant expansion in this class, with a median of approximately 24 clusters, suggesting a specialized reliance on peptide-based metabolites compared to the genus average. PKS clusters, while slightly less abundant, were maintained across species, typically ranging between 5 and 20 per genome (Fig. 3E). Interestingly, *B. pseudobassiana* displayed the highest intra-species variability in PKS content, indicating a high degree of genomic plasticity and potential for niche-specific chemical signatures within this lineage.

In contrast to the high inter-specific variation observed in NRPS and PKS counts, the abundance of Terpene (Fig. 3B) and PKS-NRP Hybrid (Fig. 3D) BGCs was notably consistent across the genus. These classes exhibited a highly uniform distribution, with the majority of species harboring a stable repertoire of 6–9 terpene clusters and 2–6 hybrid clusters per genome. Conversely, Ribosomally synthesized and Post-translationally modified Peptides (RiPPs) BGCs (Fig. 3G) were remarkably rare, often limited to one or two clusters or entirely absent, as seen in *B. asiatica*. A unique expansion in the “Others” category (Fig. 3F) was also observed in *B. pseudobassiana*, hinting at the presence of unclassified or novel biosynthetic pathways that remain to be characterized (Fig. 3). The profile of distinct BGCs in *B. bassiana* AS272 follows the general distribution of BGCs in *Beauveria* genomes, totaling 39 predicted clusters. While its genomic repertoire aligns closely with the species median for most classes, the isolate exhibits specific counts of 19 NRPS, 8 PKS, 7 Terpene, and 4 PKS-NRP Hybrid clusters (red dots in Fig. 3).

**Fig. 4:**
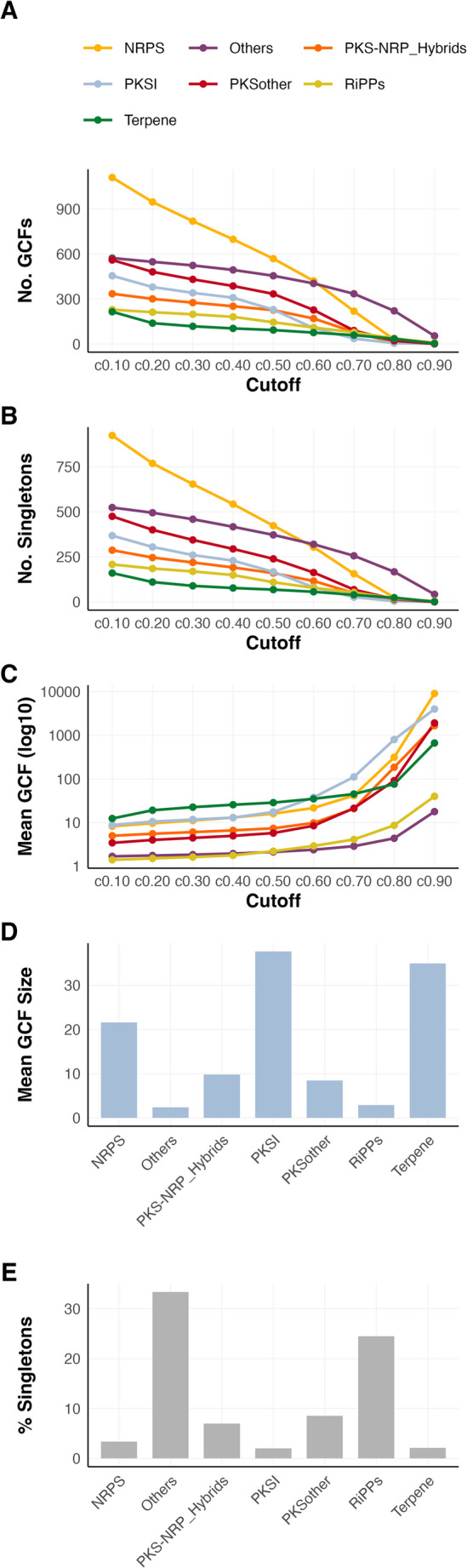
Comparative analysis of biosynthetic potential and Gene Cluster Family (GCF) dynamics across genomic datasets. (**A**) Total number of GCFs identified across a gradient of clustering stringency cutoffs (0.10 to 0.90). (**B**) Frequency of “singleton” clusters (BGCs that did not group into a family) across the same stringency gradient. (**C**) Mean GCF size (number of member clusters per family, log10 scale) as a function of clustering cutoff. (**D**) Comparison of mean GCF size at the selected cutoff (0.6) across biosynthetic classes, including Type I Polyketide Synthases (PKSI), Terpene, Non-ribosomal Peptide Synthetase (NRPS), and hybrids. (**E**) Percentage of singleton clusters at the selected cutoff (0.6) relative to the total number of BGCs within each biosynthetic category

To further investigate the evolutionary relationships between the predicted BGCs, we performed a systematic clustering analysis using BiG-SCAPE. By applying a range of sequence similarity cutoffs (0.10 to 0.90), we evaluated the conservation of BGCs into Gene Cluster Families (GCFs) across the *Beauveria* genus. As expected, the number of identified GCFs decreased significantly as the clustering threshold became more stringent (higher cutoff), indicating that many clusters share common ancestral architectures (Fig. 4A). This is reflected in the number of “singleton” BGCs—those unique to a single genome—which decreased sharply as the cutoff moved toward more inclusive similarity levels (Fig. 4B).

The mean size of the GCFs (number of BGCs per family) showed an exponential increase at higher cutoffs, particularly for the NRPS and PKS classes (Fig. 4C). This suggests that a substantial portion of the *Beauveria* specialized metabolome consists of highly related biosynthetic pathways that likely produce similar core chemical scaffolds. At a selected cutoff of 0.60, which provides a balanced resolution between over-clustering and excessive fragmentation, the PKSI and Terpene classes exhibited the highest mean GCF sizes (Fig. 4D). This indicates that these specific classes are characterized by large, multi-member families that are widely distributed across the genus. Finally, the proportion of singleton clusters varied markedly by chemical class (Fig. 4E). While PKSI, Terpene, and NRPS clusters showed a low percentage of singletons (typically < 5%), suggesting they belong to well-established, genus-wide families, the “Others” and RiPPs categories were dominated by unique, strain-specific clusters (> 25% singletons). This high degree of uniqueness in unclassified clusters highlights a reservoir of genomic novelty that may drive specific ecological adaptations or host-specialized interactions in individual *Beauveria* isolates, including the high-virulence AS272 strain.

**Fig. 5:**
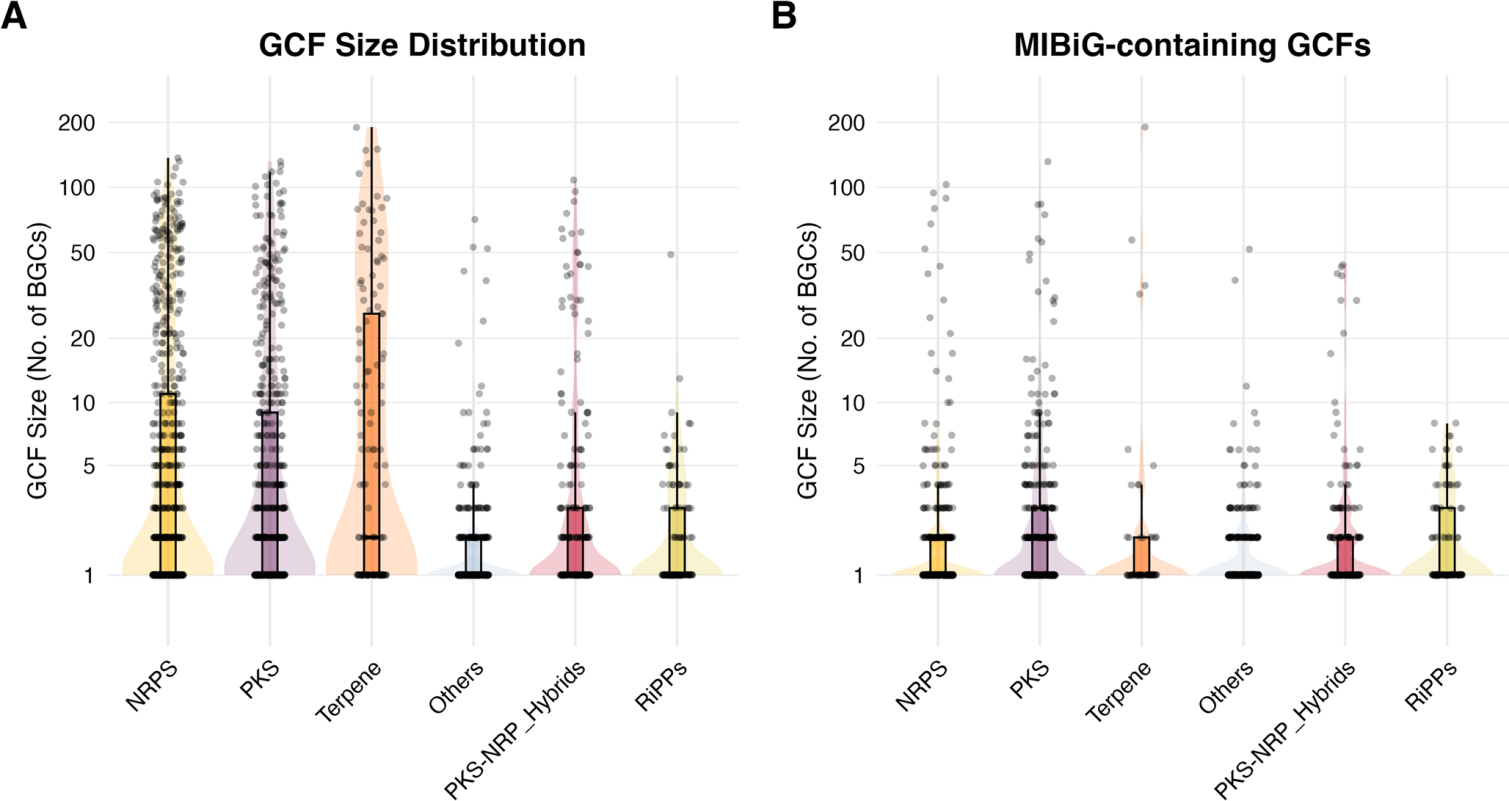
**Diversity and functional characterization of Gene Cluster Families (GCFs) across the** ***Beauveria*** **genus.** (**A**) Distribution of GCF sizes across major chemical classes. The vertical axis (log10 scale) represents the number of BGCs grouped into each family at a 0.60 similarity cutoff. (**B**) Size distribution specifically for GCFs that contain hits from the MIBiG database, representing experimentally characterized biosynthetic pathways. In both panels, violin plots illustrate the density of families, while inner box plots indicate the median and interquartile range (IQR). Individual data points represent distinct GCFs

The distribution of Gene Cluster Families (GCFs) across the genus *Beauveria* was further evaluated. The analysis of GCF size, which is defined by the number of BGCs per family, revealed distinct evolutionary patterns among chemical classes (Fig. 5). The PKSI, Terpene, and NRPS classes exhibited the most expansive family sizes, with some GCFs containing over 100 individual BGCs. This indicates that a significant portion of the *Beauveria* specialized metabolome is composed of highly related biosynthetic pathways shared by a vast majority of the 322 genomes. The largest median GCF sizes were observed in the PKSI and Terpene families, suggesting the significant importance of these specific conserved pathways to the distinct lifestyles of *Beauveria*. In contrast, the PKS-NRP Hybrids, PKSother, RiPPs, and Others categories were characterized by significantly smaller GCFs, with medians frequently falling below five members. This suggests that these classes are more prone to diversification or are restricted to specific lineages. When filtering for GCFs containing known clusters from the MIBiG database, the mean GCF size dropped across all categories, although PKSI and NRPS retained the most robust presence. This drop highlights a substantial gap in the functional characterization of *Beauveria* secondary metabolism, as the majority of large, well-conserved families currently lack a corresponding characterized metabolite in public databases.

To characterize the core biosynthetic potential of the genus *Beauveria*, a conservation analysis was performed on Gene Cluster Families (GCFs) containing at least 100 members. This threshold was selected because such a wide distribution across species and strains suggests these clusters encode for specialized metabolites that are fundamental to *Beauveria* biology. A total of 21 GCFs met this criterion, and their occupancy, defined as the percentage of species within a gene harboring the cluster, reveals distinct patterns of evolutionary stability and lineage-specific adaptation (Table S3). Several GCFs exhibited high occupancy across nearly the entire phylogeny, representing the stable biosynthetic framework of the genus. NRPS GCF 1963 and NRPS GCF 16,320 emerged as two of the most ubiquitous pathways, with 100% occupancy across *B. caledonica*,* B. medogensis*, and *B. rudraprayagi* (GCF 1963) or *B. asiatica*,* B. australis*, and *B. caledonica* (GCF 16320). PKSI GCF 7963 was equally prominent, maintaining 100% occupancy in *B. asiatica*,* B. australis*,* B. medogensis*, and *B. rudraprayagi.* Terpene GCF 12,284 demonstrated a unique distribution, present in 100% of *B. australis*,* B. brongniartii*,* B. caledonica*,* B. medogensis*, and *B. rudraprayagi* isolates, despite being nearly absent in *B. bassiana* (2%) and *B. pseudobassiana* (0%).

In contrast to the ubiquitous core, several large GCFs served as definitive markers for specific clades. For *B. bassiana* and *B. rudraprayagi*, Terpene GCF 1989 showed high frequency in *B. bassiana* (85.9%) and 100% occupancy in *B. rudraprayagi*, but was absent in most other lineages. The sister species *B. pseudobassiana* and *B. varroae* share a unique biosynthetic profile, defined by 100% occupancy of NRPS GCF 16,292, PKSI GCF 16,319, and PKSother GCF 4094. Furthermore, NRPS GCF 15,871 and NRPS GCF 19 showed nearly universal presence in these two clades (> 95%) while remaining restricted from the rest of the genus.

The conservation analysis also highlighted GCFs with hits in the MIBiG database, providing insight into the chemical nature of these core clusters. Terpene GCF 1989 (linked to BGC0001839.1) and PKSI GCF 7963 (linked to BGC0001720.1) represent well-characterized pathways widely distributed across the genus. Notably, NRPS GCF 19 was associated with multiple MIBiG entries (BGC0000959.1, BGC0001136.1, and BGC0002035.1), suggesting it may represent a cluster family responsible for a known class of bioactive compounds like beauvericin or bassianolide.

Within the *B. bassiana* complex, including the isolate AS272, occupancy for these 21 large GCFs typically fluctuated between 40% and 60%. This moderate frequency reflects the significant intra-specific genomic plasticity of the lineage. To characterize the species-specific biosynthetic landscape, we first attempted to identify a strictly conserved set of Gene Cluster Families (GCFs) within *B. bassiana*. Despite being the most abundant species in the dataset, comprising 199 of the 322 genomes analyzed, the results revealed that *B. bassiana* possesses zero strictly core GCFs (defined by an absolute 100% occupancy threshold across all isolates).

**Fig. 6:**
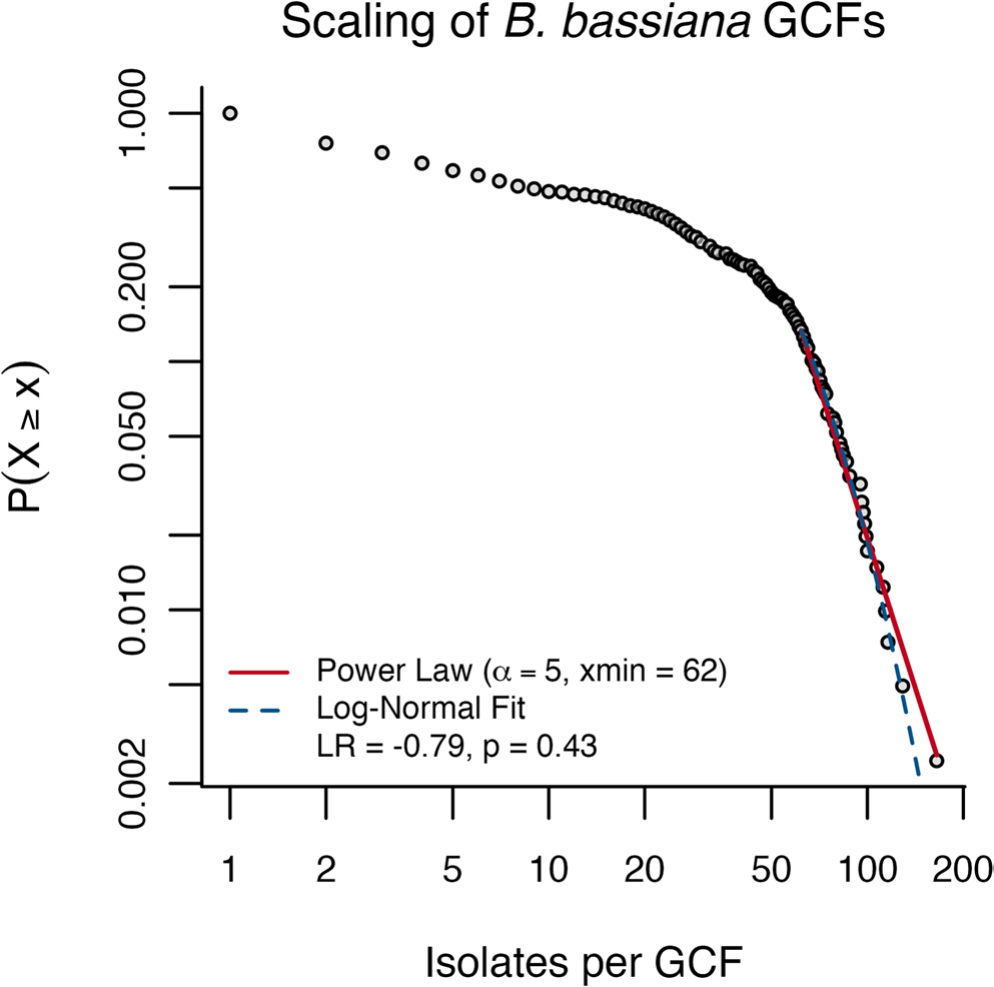
**Power Law scaling and genomic fluidity of** ***B. bassiana*** **Gene Cluster Families (GCFs).** The log-log plot displays the Complementary Cumulative Distribution Function (CCDF), defined as P(X ≥ x), which represents the probability that a given GCF is shared by at least x number of isolates. The empirical data (open circles) were modeled using a Power Law distribution (solid red line) characterized by a scaling exponent α = 5.41 and a lower threshold x_min_ = 71. A Log-Normal fit (dashed blue line) was overlaid for comparison. A Vuong likelihood ratio test (LR = − 0.61,*p* = 0.543) indicates that neither model provides a significantly better fit, suggesting the distribution of GCFs follows a heavy-tailed scaling behavior across the *B. bassiana* isolates

Given this observation, we evaluated the distribution of biosynthetic gene cluster families (GCFs) exclusively within the *B. bassiana* lineage using discrete power-law and log-normal modeling to investigate how these clusters are maintained across isolates. The analysis revealed a highly skewed distribution, with substantial variance in GCF occupancy among isolates (Fig. 6). However, model comparison using the Vuong likelihood ratio test did not support a significantly better fit of the power-law model over the log-normal alternative (LR = − 0.61, *p* = 0.543). Although a power-law fit yielded an estimated scaling exponent of α = 5.41 (x_min_ = 71), the statistical results do not support a preference for either model. Overall, the analysis indicates that a relatively small number of GCFs are widely conserved across isolates, whereas most occur at lower frequencies, highlighting the substantial genomic and biosynthetic diversity within *B. bassiana* (Fig. 6).

While no strictly core clusters were found, the modeling identified some GCFs, which represent high-frequency clusters that could form the functional backbone of the species’ chemical repertoire without being universally present in every strain. The occupancy data for these hubs further illustrates this flexibility. The most prevalent family, Terpene GCF 1989, reached only 85.9% occupancy, followed by Terpene GCF 8171 (67.3%) and NRPS GCF 8144 (59.3%). Other major families such as PKSother 12,262 (57.8%) and PKSI 8173 (56.8%) also displayed moderate to high frequency but remained absent in a significant portion of the population. Within the *B. bassiana* complex, including the high-virulence isolate AS272, occupancy for these 21 large GCFs typically fluctuated between 40% and 60%. This moderate frequency, compared to the strict 100% occupancy seen in smaller clades, reflects the significant intra-specific genomic plasticity of the lineage. This suggests that while *B. bassiana* maintains a core set of BGCs, it utilizes a more variable set of high-frequency clusters to potentially tailor its pathogenic profile to specific hosts or environmental niches.

### Association of ***B. bassiana*** AS272 BGCs with virulence

To investigate the functional context of conserved biosynthetic gene clusters and their potential association with pathogenicity, we performed a protein–protein interaction (PPI) network analysis. Interaction networks were constructed for the core genes of six highly conserved hub GCFs using the STRING database (version 12.0), based on the *B. bassiana* AS272 full proteome annotation. For this analysis, we selected GCFs with high prevalence across the genus (≥ 90 members), representing five biosynthetic classes: Terpene, NRPS, PKS–NRP hybrids, PKS-like, and T1PKS.

**Fig. 7:**
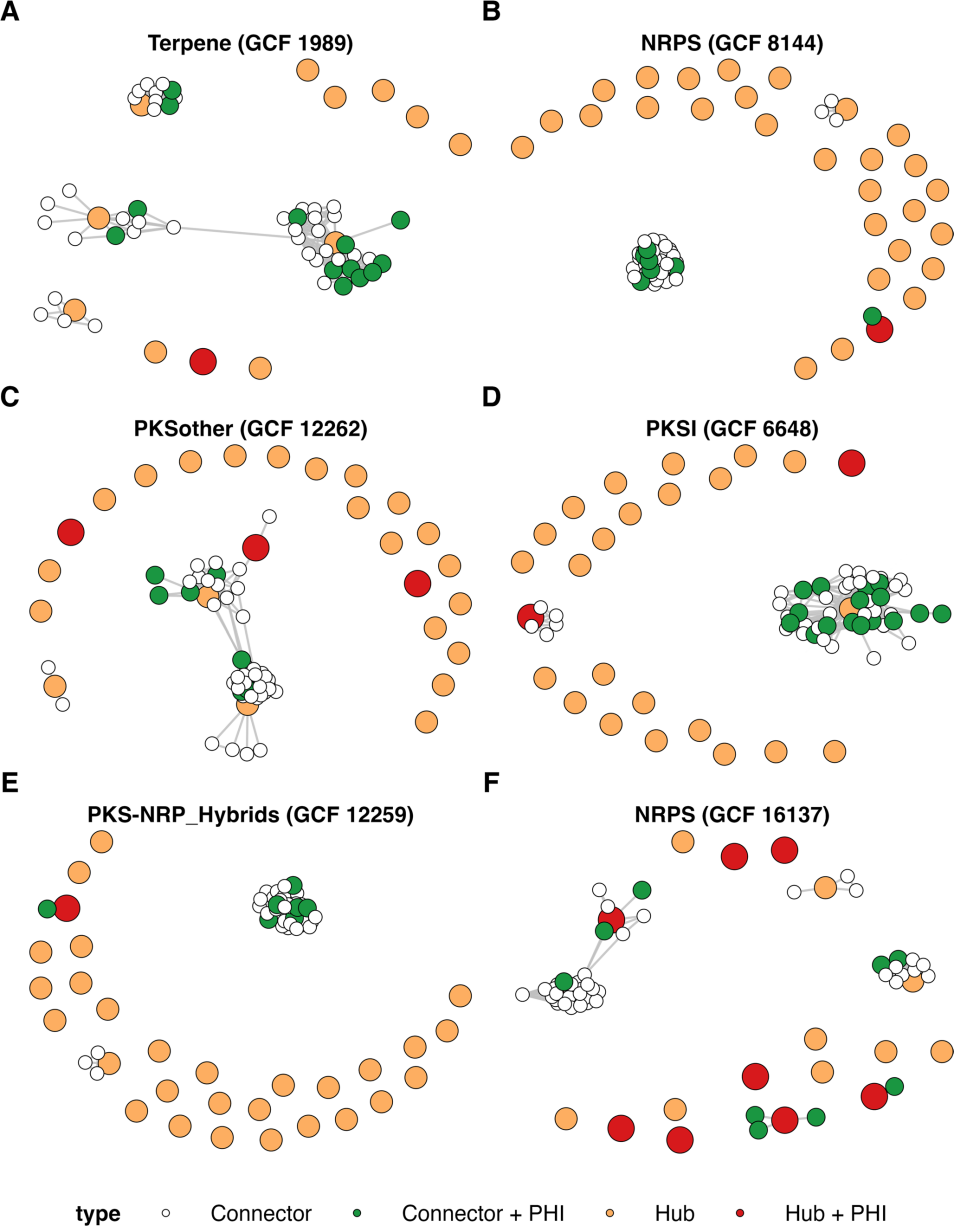
Protein-Protein Interaction (PPI) Networks of Conserved Biosynthetic Gene Clusters (BGCs). Functional interaction landscapes for the six most prevalent Gene Cluster Families (GCFs) in the *Beauveria* genus, centered on the *B. bassiana* AS272 proteome. (**A**–**F**) Networks represent individual hub GCFs containing at least 90 members. Central hub nodes (circles with solid borders) represent biosynthetic enzymes coded by BGCs and cluster-defined proteins, while peripheral connector nodes represent predicted interacting partners. Node coloring indicates pathogenicity associations derived from PHI-base cross-referencing: red nodes are BGC-encoded virulence factors; green nodes are interacting partners with known roles in pathogen-host interactions; and white/orange nodes represent proteins with no characterized PHI-base homology. Line thickness (edges) corresponds to the STRING database confidence score for the interaction (> 800)

Topological analysis indicated that these GCFs are embedded within highly interconnected interaction networks (Fig. 7). The resulting networks displayed substantial structural complexity, with an average node degree ranging from 15.23 (Terpene GCF 1989) to 43.75 (NRPS GCF 8144). A notable feature of these networks is their high clustering coefficients, ranging from 0.58 to 0.95, which exceeded expectations for comparable random networks. Correspondingly, clustering enrichment values ranged from 1.55 to 2.85, indicating a dense and modular organization in which biosynthetic core genes relate to broader cellular interaction networks (Table S4).

To assess potential associations with pathogenicity, network proteins were cross-referenced against the PHI-base database to identify homologs of experimentally characterized pathogen–host interaction factors. The results of comparative analysis of the predicted proteome of *B. bassiana* AS272 against PHI-base are presented in Table S5, while the identity of *B. bassiana* AS272 BGC genes related to the hub GCFs is present in Table S6. Across the six networks, the number of PHI-related nodes ranged from 8 to 18 (Fig. 7). Statistical enrichment analysis indicated that these associations are unlikely to arise by chance. In particular, the NRPS (GCF 8144) and PKS–NRP hybrid (GCF 12259) networks showed the strongest enrichment for known virulence-associated proteins (FDR-adjusted *p* = 1.90 × 10⁻⁶). The PKSother network (GCF 12262) also exhibited significant enrichment (*p* = 6.40 × 10⁻⁵). Although the Terpene and T1PKS networks contained multiple PHI-base homologs (14 and 18 nodes, respectively), their enrichment did not reach the same level of statistical significance (FDR > 0.05). This pattern may reflect interactions with a broader or less well-characterized set of host-associated factors. Taken together, these results suggest that some conserved biosynthetic gene clusters in *Beauveria*, particularly NRPS and hybrid PKS–NRP systems, are embedded within interaction networks enriched for proteins associated with host interaction and pathogenicity (Tables S5 and S6).

## Discussion

Analysis of the *B. bassiana* isolate AS272 reveals a genomic architecture geared toward both metabolic versatility and pathogenic potential. While some studies emphasize the contribution of lineage-specific genes (singletons) to virulence, our results suggest that the aggressiveness of this isolate may instead be associated with an expansion of conserved gene families. The relatively low proportion of orphan genes compared to orthogroups indicates that the AS272 genome has likely evolved through gene duplication events, leading to increased functional redundancy and potential metabolic flexibility.

A prominent feature of the genome is the expansion of transporters belonging to the Major Facilitator Superfamily (MFS) and ATP-binding cassette (ABC) superfamilies. These transporters are known to play key roles in toxin secretion during infection and may also contribute to protection against insect-derived defense metabolites and environmental stressors (Valero-Jiménez et al. 2016). In AS272, the increased copy number of genes such as K08192 suggests a potentially enhanced efflux capacity, which may facilitate survival and colonization within host environments (Solano-González et al. 2023).

From a phylogenetic perspective, the phylogenomic reconstruction of 337 genomes revealed persistent misidentifications in public databases. The lack of clustering of strain MBC618, together with the isolated position of *B. felina*, highlights the limitations of traditional taxonomy based solely on morphology or single-locus markers such as the ITS region. Furthermore, the placement of *B. rudraprayagi* within the *B. bassiana* clade suggests that it may represent a variant or closely related lineage of *B. bassiana*, supporting previous studies that have emphasized the need for systematic revision within the *Beauveria* genus (Rehner et al. 2011). The use of single-copy orthologs identified through the BUSCO framework (Manni et al. 2021) provides a robust phylogenomic basis for species identification and for the accurate documentation of strains used in industrial or biotechnological applications.

Another notable finding is the apparent absence of a strictly conserved core of biosynthetic gene clusters (BGCs) within *B. bassiana*. Instead, the distribution of these clusters follows a highly dynamic pattern, potentially enabling the fungus to maintain a flexible chemical arsenal and considerable genomic plasticity (Valero-Jiménez et al. 2016). While certain classes, such as terpene clusters, show relatively high conservation—suggesting roles in core biological processes—NRPS and PKS clusters appear to function primarily as sources of chemical innovation. Such intraspecific variation may explain the substantial differences in virulence observed among *B. bassiana* strains infecting the same host species, as individual strains may deploy distinct combinations of secondary metabolites derived from diverse biosynthetic hubs (Toopaang et al. 2025).

Protein–protein interaction (PPI) network analysis further suggests that secondary metabolite biosynthesis is integrated within broader pathogenicity-related networks. Cross-referencing these networks with PHI-base identified several connections between core BGC proteins and previously described virulence-associated factors, indicating potential coordination between toxin biosynthesis and infection-related processes such as cuticle degradation. Although compounds such as beauvericin and oosporein are known to play important roles in suppressing host immune responses, most of the identified GCFs lacked direct counterparts when compared against the MIBiG database (Zdouc et al. 2025).

The dynamic distribution of BGCs observed across *B. bassiana* isolates likely reflects the rapid evolutionary turnover typical of fungal secondary metabolite pathways. Statistically, the distribution of GCF occupancy, which is equally well described by log-normal and power-law models, suggests that BGC retention is not governed by a single-mechanism scale-free process, such as pure preferential attachment. Rather, this distribution is indicative of a mixture of independent, compounding evolutionary pressures. Biosynthetic clusters are frequently shaped by gene duplication, recombination, horizontal transfer, and lineage-specific gene loss, processes that collectively generate substantial chemical diversity within species. Such evolutionary plasticity allows entomopathogenic fungi to adapt to diverse ecological niches and host environments by modifying or expanding their repertoire of bioactive compounds. In the case of *Beauveria*, this flexible biosynthetic architecture may provide a selective advantage during host infection, where different insect species present distinct physiological defenses. Consequently, the diversification and differential retention of BGCs may represent an important evolutionary strategy enabling *B. bassiana* populations to maintain pathogenic versatility across a broad range of insect hosts.

The absence of clear matches in MIBiG underscores the largely unexplored biosynthetic diversity within the *Beauveria* genus. This hidden repertoire of specialized metabolites may represent a substantial source of novel bioactive compounds with potential applications in biotechnology, agriculture, and pharmaceutical discovery, warranting further functional and chemical characterization.

## Supplementary Information

Below is the link to the electronic supplementary material.

Supplementary Material 1 (CSV 732 bytes)

Supplementary Material 2 (CSV 589 bytes)

Supplementary Material 3 (CSV 22.5 KB)

Supplementary Material 4 (CSV 614 bytes)

Supplementary Material 5 (CSV 15.9 KB)

Supplementary Material 6 (R 45.6 KB)

Supplementary Material 7 (CSV 196 KB)

Supplementary Material 8 (SH 12.6 KB)

Supplementary figure 8 (PNG 1.32 MB)

High Resolution Image (TIF 2.47 MB)

## Data Availability

The codes used for shell processing, as well as for R analysis and figures plotting, are available as Supplementary Files 1 and 2, respectively. Processing files are also available ate ZENODO 10.5281/zenodo.20672312.
