# Correction to: Genome-wide exploration of biosynthetic gene clusters and their association with virulence in the entomopathogenic fungus *Beauveria*

**DOI:** 10.1007/s10142-026-01976-z

**Published:** 2026-07-10

**Authors:** Robson dos Santos Soares, Alexandra de Azevedo da Rocha, Matheus Camargo, Maria Eduarda Deluca João, Augusto Schrank, Henrique R. M. Antoniolli, Charley Christian Staats

**Affiliations:** 1 Programa de Pós-Graduação em Biologia Celular e Molecular, Centro de Biotecnologia, Universidade Federal do Rio Grande do Sul, Av. Bento Gonçalves 9500, Porto Alegre, 91501-970 Agronomia, RS Brasil; 2 National Institute of Science and Technology in Human Pathogenic Fungi, São Paulo, Brazil

**Correction to: Functional & Integrative Genomics**

10.1007/s10142-026-01958-1

In the original version of this manuscript, the co-author who was designated as a shared corresponding author was not correctly identified in the final publication.

The shared corresponding author should be Dr. Henrique R. M. Antoniolli (antoniolli.henrique@hotmail.com).

The original article has been corrected.

