# Supplementary figures and images for: Genome-wide exploration of biosynthetic gene clusters and their association with virulence in the entomopathogenic fungus Beauveria
Source: Funct Integr Genomics. 2026 Jun 30;26(1):170. doi: 10.1007/s10142-026-01958-1 (PMC13314832; doi:10.1007/s10142-026-01958-1)

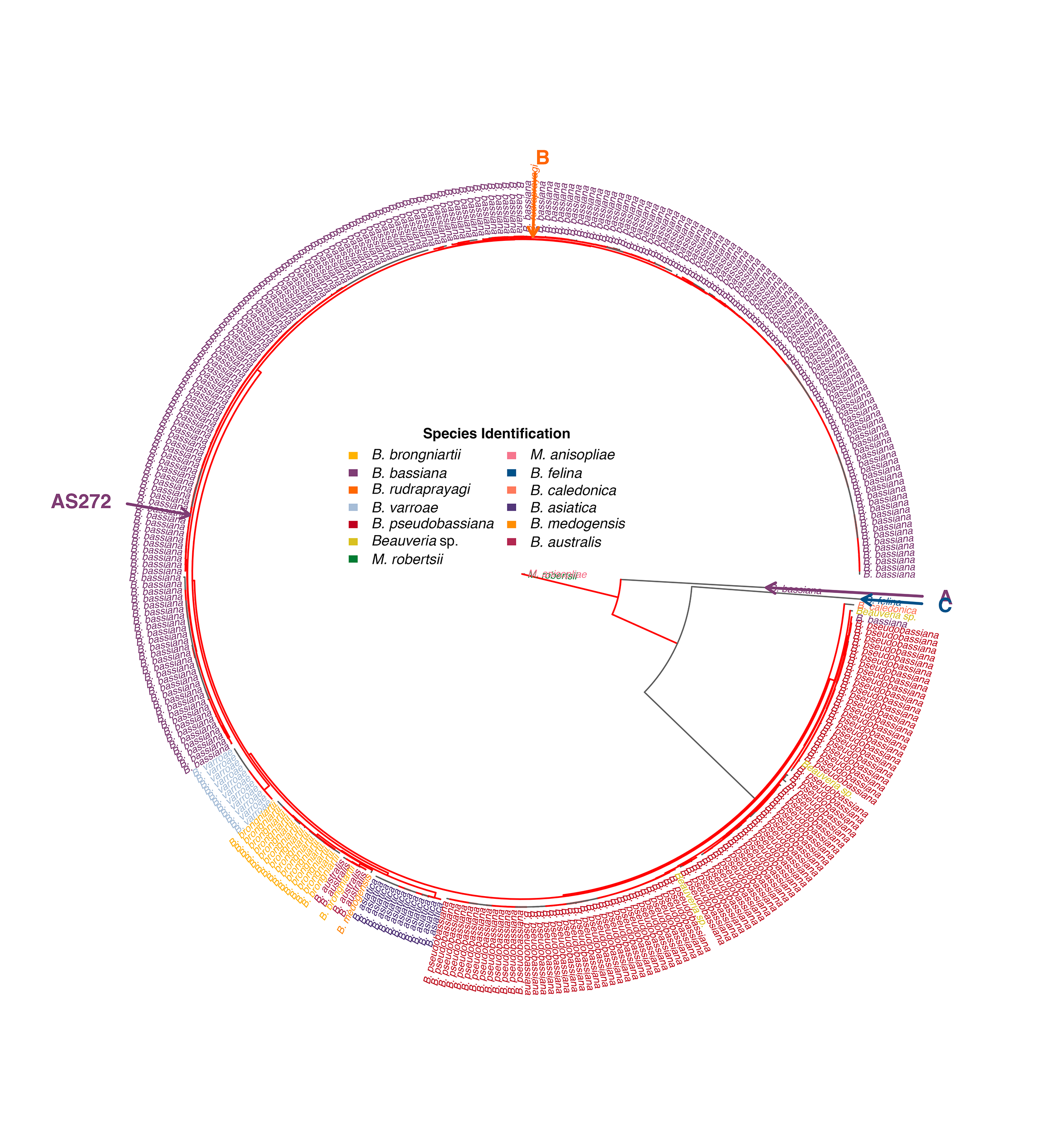

Supplement: Supplementary file 9 — (PNG 1.32 MB) [file 10142_2026_1958_Fig8_ESM.png]

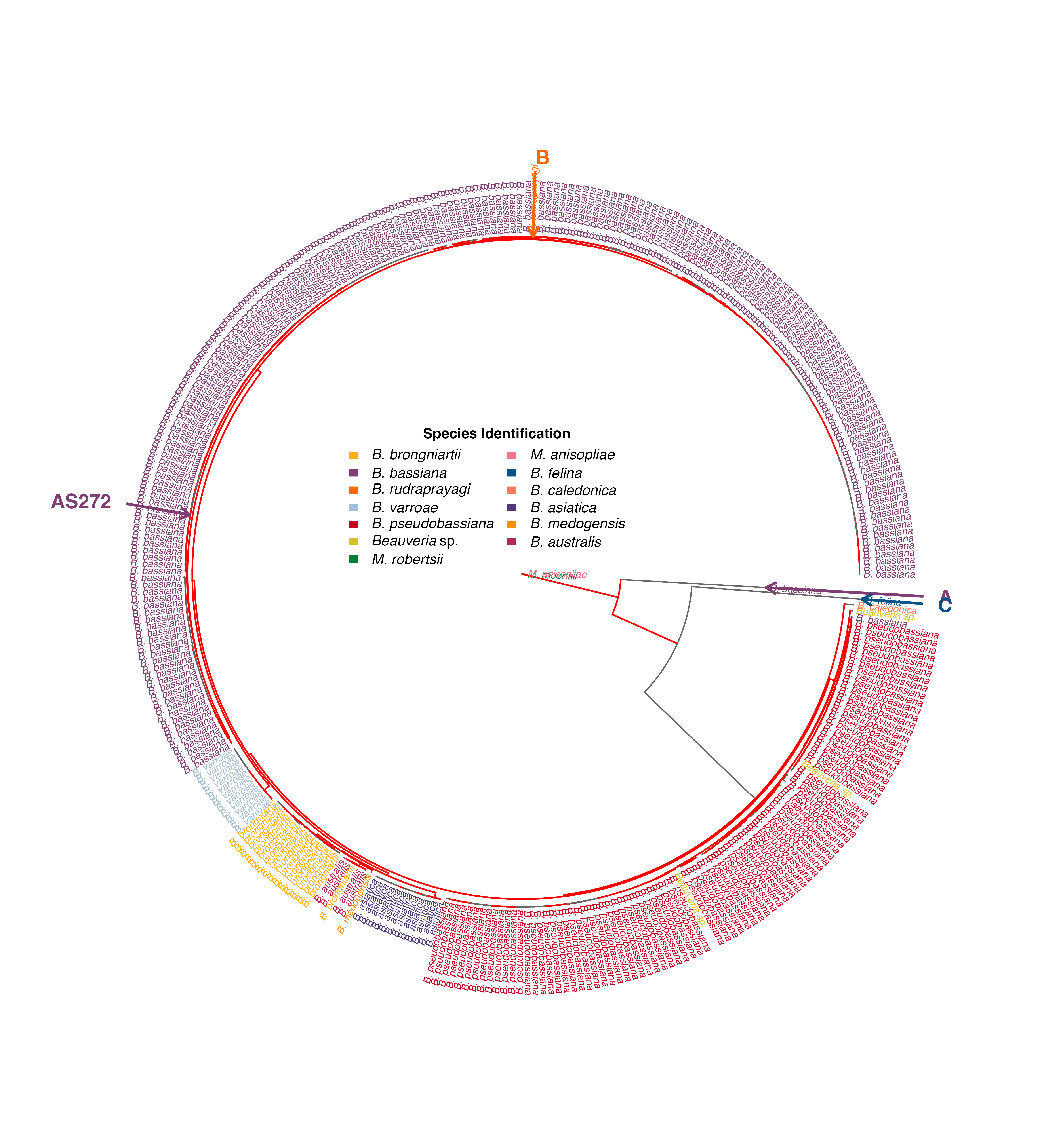

Supplement: Supplementary file 10 — High Resolution Image (TIF 2.47 MB) [file 10142_2026_1958_MOESM9_ESM.tiff]
